# Two cases of atypical twinning: Phenotypically discordant monozygotic and conjoined twins

**DOI:** 10.1002/ccr3.2113

**Published:** 2019-03-29

**Authors:** Maria E. Barnes‐Davis, DonnaMaria E. Cortezzo

**Affiliations:** ^1^ Department of Pediatrics University of Cincinnati College of Medicine Cincinnati Ohio; ^2^ Division of Neonatal and Pulmonary Biology Cincinnati Children’s Hospital Medical Center Cincinnati Ohio; ^3^ Department of Anesthesiology University of Cincinnati College of Medicine Cincinnati Ohio; ^4^ Division of Pain and Palliative Medicine Cincinnati Children’s Hospital Medical Center Cincinnati Ohio

**Keywords:** assisted reproduction technologies, conjoined, discordant, monozygotic, twinning

## Abstract

Atypical twinning highlights that complex mechanisms responsible for twinning are not fully understood and may give further insight into the mechanisms involved. To assume that phenotypic difference is the result of dizygotic twinning would be erroneous and could have significant implications in the care and counseling provided to these patients.

## 1 INTRODUCTION

We report two cases of atypical monozygotic twins to provide insight into embryogenesis. They are discussed within the fission and fusion concepts of twinning. Epigenetic mechanisms that may contribute to pathogenesis of discordant monozygotic twins are reviewed. These concepts are important for the counseling and care of atypical twins.

Monozygotic (MZ) twinning is a rare event in nature, occurring in humans and armadillos.^1^ While dizygotic (DZ) twinning rates vary throughout the world, monozygotic rates were previously stable at <0.5% of pregnancies.^1^, ^2^ Twin births, both DZ and MZ, are becoming increasingly common.^2^, ^3^ This is attributed to advanced maternal age and assisted reproduction technologies (ART), most commonly associated with in vitro fertilization (IVF).^4^, ^5^ While increased rates of twinning were historically associated primarily with transfer of multiple embryos during IVF or release of multiple oocytes from ovarian stimulation, recent evidence and experience suggest that ART with single embryo transfers also results in increased MZ twinning. Blastocyst transfer, intracytoplasmic sperm injection, storage or manipulation of the embryo, and abnormal X inactivation may contribute to increased rates of MZ twins.^1^, ^5^, ^9^, ^10^ Embryo transfer at the blastocyst stage is most commonly associated with MZ twinning in case‐control studies and meta‐analyses; nevertheless, it is likely that techniques employed in ART/IVF have synergistic effects.^5^, ^11^, ^12^ ART has been reported in association with atypical twinning.^17^, ^18^ Several reports challenge the classical fission theory of twinning and highlight that the mechanisms resulting in MZ twinning remain poorly understood.^5^, ^14^, ^17^, ^20^, ^21^

Previously, fission (embryo splits into two distinct entities, membrane anatomy dependent on timing of separation) was believed to explain all MZ twinning. According to the most widely accepted theory, fission occurs 1‐3 days after fertilization in dichorionic diamniotic twins, 4‐6 days in monochorionic diamniotic twins, and 7‐9 days in monochorionic monoamniotic twins. Partial fission after the development of the primitive streak at approximately 2 weeks results in conjoined twins.^1^, ^2^, ^21^ However, fission has never been observed in vitro or in vivo.^20^ Well‐documented cases of MZ twins with variable chorionicity and amnionicity occurring after single embryo transfer at the blastocyst stage (5‐7 days) and of dizygotic monochorionic twins after ART are incongruent with fission.^11^, ^14^, ^17^ Fission fails to account for several forms of atypical twinning, including chimeric, acardiac, asymmetrically conjoined, and fetus in fetu. In these instances, fusion theory—where the embryo refuses after two‐cell postzygotic splitting with placentation and membranes dependent on degree of fusion of inner cell masses, trophoectoderm, etc—might be more congruent.^21^

In this report, we describe two pairs of phenotypically discordant MZ twins, one of which are conjoined. We review relevant literature with the aim of discussing theories of twinning and the impact of ART on incidence and pathogenesis of discordant twinning.

## 2 CASE REPORT

Twin Pair 1 were spontaneously conceived monochorionic, monoamniotic MZ males with 19% growth discordance born at 26 weeks to a Gravida 3 Para 2→4 mother via C‐section for breech presentation, decelerations, and reversed end‐diastolic flow. Twin A transferred to our hospital at 3 months of age for severe chronic lung disease, pulmonary hypertension, and airway evaluation necessitating tracheostomy. He had absent left kidney, prenatal MRI showing polymicrogyria, and postnatal cranial ultrasound showing prominent lateral ventricles, cystic periventricular leukomalacia, and intraventricular hemorrhage detected after new‐onset seizures. He developed retinopathy of prematurity requiring treatment. Radiographs revealed 13 rib pairs and suspected vertebral anomalies. Physical examination was notable for short limbs. Twin B had less‐severe lung disease and retinopathy of prematurity requiring no intervention. He had no renal or neurological abnormalities. At time of Twin A's transfer, Twin B was home on full feeds and nasal cannula. Karyotype, microarray, and zygosity testing were performed, demonstrating monozygosity and concordant deletion of Yq11.22 not felt to be responsible for Twin A's phenotype.

Twin Pair 2 were spontaneously conceived, conjoined thoraco‐omphalopagus, monochorionic, monoamniotic, MZ females born at 31 weeks via C‐section to a Gravida 2 Para 0→2 mother with preeclampsia. On MRI, twins shared a liver with separate portal venous and biliary systems confirmed at operative separation. They shared a single 6‐vessel umbilical cord and had discordant cardiac anatomy. Twin A had balanced complete atrioventricular canal (CAVC). Twin B had normal cardiac structure. They were conjoined from xiphoid to pubic symphysis, resulting in face‐to‐face configuration. Each had intact genitalia, anuses, and lower limbs. Tissue expanders were placed at 1.5 months of age, with successful separation at 3 months. Twin A underwent repair of her CAVC. She was discharged from the CICU at 9 months on supplemental oxygen via nasal cannula and nasojejunal feeds. Twin B had a shorter stay with hematochezia and feeding intolerance which resolved before discharge at 6 months of age.

## 3 DISCUSSION

### 3.1 Biology of atypical twinning

We present two MZ pairs with remarkable phenotypic discordance despite their monozygosity to highlight relevant theories about this complex phenomenon in reproductive medicine. While precise mechanisms causing discordance are unknown, genetic (chromosomal, single gene defects, epigenetics including differential methylation and imprinting, mitochondrial, skewed X inactivation) and environmental (cell number at division, vascularity, placental attachment, embryonic signaling, postnatal experiences) factors might contribute to distinct features of MZ twins.^1^, ^2^, ^21^, ^23^, ^24^ Discordant twinning is rare, occurring in approximately 10% of MZ twin pairs. There is limited knowledge regarding pathogenesis.^1^ Embryogenesis is complex and incompletely elucidated, involving iterative processes with signaling molecules, centers, and morphogenetic gradients.^25^ Aberrant cross‐signaling could occur when MZ twins have conflicting migrational or morphogenetic pathways resulting in conjoined twins or phenotypic discordance.^26^ With the exception of humans and armadillos (the latter birth identical quadruplets), monozygosity is not seen in other placental mammals. Experimentally, MZ twinning has been induced in other species by disruption of the zone pellucida, zona tampering (in which integrity is breached by assisted hatching, ICSI, etc, leading to damage or splitting of the cell mass), delayed fertilization, and exposure to hypoxia.

### 3.2 Fission vs fusion

Atypical twins provide unique insights into theories of fission vs fusion. Conjoined twins—discordant or not—can present a unique challenge to fission theory. They are obligate MZ occurring only 1 in 250 000 live births, or 1 in 400 MZ twin pairs.^1^, ^2^, ^27^ Within the fission framework, these twins are the result of incomplete separation of embryonic plates. Blastogenesis of conjoined twins is still not understood.^26^ Existence of asymmetric and acardiac twins is incongruent with fission theory, which might make fusion mechanisms more likely in these scenarios. In this theory, MZ twins could undergo fusion at a later point in the development beyond zygotic splitting. The triggers for fusion are unknown, but theories include like stem cells being attracted to each other or a reorganization of such cells after two distinct axes have been recognized.^20^ Twin Pair 2 had symmetric thoracic attachment, but the phenotypic discordance might support fusion over fission.

### 3.3 Role of epigenetics

Divergent epigenetic expression likely contributes to distinct growth and development in MZ twins.^5^ Epigenetics refers to inheritance of gene expression patterns not based on DNA sequence. Events occurring in utero influence fetal growth and metabolism and have life‐long consequences, epitomized by concepts related to the “Barker hypothesis.”^28^ Intrauterine environmental factors (such as the proportion of the placenta, placental blood flow, and umbilical cord supporting each cotwin) may differentially affect each twin resulting in distinct developmental and metabolic processes. Gene expression is strongly influenced by changes in chromatin and differences in methylation and histone acetylation. Epigenetic markers such as DNA methylation in MZ twins change as distinct postnatal experiences diverge. DZ twins have more epigenetic discordance than MZ twins.^29^ Differences in placentation, site of implantation, cord insertion, etc, may confer changes in maternofetal blood flow, resulting in differences in substrate and oxygen supply which may in turn contribute to epigenetic variation.^29^, ^30^ These changes are thought to underlie diseases later in life, including neoplastic, autoimmune, and psychiatric disease.^30^, ^31^ Genomic imprinting related to differential methylation of paternal and maternal alleles was identified in cases of discordant Beckwith‐Wiedemann and Silver Russell in MZ twins.^2^, ^32^, ^36^ Changes in gene expression or growth of embryonic progenitor cells may have profound changes in tissue and organ morphogenesis; for example, increased methylation at the AXIN1 gene was implicated in discordant caudal duplication syndrome in MZ twins.^33^ Mechanisms such as these likely underlie some phenotypic discordance in MZ twins regardless of which theoretical pathway—be it fission or fusion—initiated the twinning event.

### 3.4 Postzygotic differences

Genetic bases for discordant MZ twins include postzygotic differences, such as chimerism, somatic cell mosaicism, X inactivation, spontaneous single gene defects, or copy number variants. Differences in chromosome inactivation are implicated in female MZ twins discordant for Duchenne muscular dystrophy, Turner syndrome, hemophilia, Hunter's disease, and Fabry's disease.^37^, ^38^ Female predominance of conjoined twins and association of phenotypic discordance of X‐linked diseases with skew in MZ twin pairs led to speculation that X inactivation influences pathogenesis, but this was not demonstrated in tested cases.^41^ Postzygotic genetic changes have been reported for mosaic twins (abnormal twin with minor anomalies found to have 46XX dup(1)(p12p15)), twins with increased copy number variation, and de novo mutations in discordant twins causing neurofibromatosis 1 and Dravet's syndrome.^42^, ^43^ In Dravet's syndrome (also known as severe myoclonic epilepsy of infancy), exome sequencing of concordant and discordant twin pairs demonstrated mutation of the SCN1A gene (encoding a sodium channel subunit) could occur at any time in the life cycle of the gene through single gene mutation in the gamete or mosaicism of somatic cells or germ line cells, with each class of mutation having different significance for reproductive counseling.^42^ However, in some discordant pairs, no nucleotide sequencing differences were found by next‐generation sequencing. Conversely, copy number and point mutations were identified in concordant adult MZ twins; however, the estimated mutation rate of 1.2 × 10^−7^ per base pair per twin pair makes the biological significance of these nucleotide differences unclear.^44^, ^48^, ^49^ While these reports do not provide evidence specifically for fission or fusion, they underscore the complexity of the biological milieu surrounding the twinning event—or sequence of events—and subsequent development of the cotwins.

### 3.5 Diagnosis

Understanding zygosity and mechanisms of twinning has implications for genetic counseling, risk stratification, and medical issues in childhood and beyond. The presented cases highlight diagnostic and prognostic challenges faced by caregivers and families prenatally and postnatally. It is important that providers do not rely on placentation, chorionicity, or amnionicity under the assumption of fission theory for zygosity determination, which generally relies on examination of the mother and findings from ultrasound.^50^, ^51^ Membrane anatomy is used as surrogate for zygosity to predict congenital defects and need for genetic testing, but is not reliable.^50^ Indeed, a recent report of monochorionic twins—assumed to be MZ—who were found to be discordant for sex underscores we cannot continue to rely on membrane anatomy for definitive diagnosis (this particular twin pair was determined to be sesquizygotic, resulting from one oocyte and two spermatozoa).^22^ Noninvasive prenatal genetic testing using cell‐free DNA can be considered, but is challenging in multiple gestations.^53^ Obstetricians and pediatricians must be aware of these limitations. Accurate genetic determination of zygosity is important for both prenatal (congenital malformation) and postnatal (hereditable disease, organ transplantation, and blood transfusion) risk stratification. The perception that MZ twins are “identical” can obscure important biological differences. Postzygotic genetic and epigenetic changes provide mechanisms that may have distinct consequences in each cotwin. Knowledge of diverse outcomes in MZ twins should impact conversations among providers and encourage caution when interpreting “twin studies.” Precise diagnosis of zygosity is important to families, who report psychological harm if later testing is inconsistent with prenatal diagnosis.^54^

### 3.6 Recommendations

Given costs and benefits of zygosity determination, we suggest: (a) Twin families have thorough medical history—including assisted reproduction and discordant features of twins—and examination. (b) Diagnostic imaging beyond standard screening should be employed on suspicion of disease. (c) Zygosity should not be assumed based on sex, placentation, or membrane anatomy. (d) Formal zygosity testing (microsatellite testing or polymerase chain reaction testing at highly polymorphic loci in cotwins and parents) is not recommended for all twins. As tests become more affordable and clinical implications more fully understood, this may change. (e) We recommend postnatal zygosity testing in the presence of perinatal or congenital disease, monochorionic infants, concordant sex, twins requiring transfusion or transplant, twins born via ART, and when patients desire accurate knowledge of zygotic identity. Zygosity testing can impact clinical care with regard to prenatal test results, reproductive counseling of parents, postnatal care of infants, and trust among families and physicians.

## 4 CONCLUSIONS

The presence of phenotypic differences in so‐called identical twins is not uncommon and has been the subject of “nature vs nurture” discourse in both the lay and scientific press. These cases, while they do not prove one theory of twinning, impress upon the clinician that these mechanisms are still not fully understood, are incredibly complex, and are multifactorial. Therefore, to assume that phenotypic difference—even something as involved as discordant caudal duplication^33^ or presence of discordant CNS malformations as we report—must be the result of dizygotic twinning would be erroneous and could have significant implications in the care and counseling provided to these patients. In such cases, consultation with a geneticist and pre‐ or postnatal testing might be warranted. Through continued reporting and examination of such cases, we might work toward a better understanding of the mechanisms of twinning and implications for human disease and health.

## CONFLICT OF INTEREST

None declared.

## AUTHOR CONTRIBUTIONS

Maria Barnes‐Davis, MD/PhD: Conceived of and produced the first draft of this manuscript and approved of the manuscript in its current form. DonnaMaria Cortezzo, MD: Provided substantive guidance and feedback on conceptulization of the  manuscript, content review, drafts of the manuscript, and submitted the report in its current form.

## DECLARATION

As this is a case report, it does not meet criteria for human subject research, and thus, approval by the Institutional Review Board and written informed consent are not required. The local practice, though, is that when a rare diagnosis is involved or that there is a reasonable likelihood that patients could be identified, that family is contacted to inform them of the intent to include the patient in a case report.

## References

[1] Hall JG . Twinning. Lancet. 2003;362(9385):735‐743. 12957099 10.1016/S0140-6736(03)14237-7

[2] Weber MA , Sebire NJ . Genetics and developmental pathology of twinning. Semin Fetal Neonatal Med. 2010;15(6):313‐318. 20663725 10.1016/j.siny.2010.06.002

[3] Hamilton B , Martin J , Osterman M , et al. Births: final data for 2014. Natl Vital Stat Rep. 2015;64(12):920‐64. 26727629

[4] Martin J , Osterman M . Three decades of twin births in the United States, 1980–2009. NCHS Data Brief. 2012(80):920‐8. 22617378

[5] Aston KI , Peterson CM , Carrell DT . Monozygotic twinning associated with assisted reproductive technologies: a review. Reproduction. 2008;136(4):377‐386. 18577552 10.1530/REP-08-0206

[6] Diamond MP , Legro RS , Coutifaris C , et al. Letrozole, gonadotropin, or clomiphene for unexplained infertility. N Engl J Med. 2015;373(13):1230‐1240. 26398071 10.1056/NEJMoa1414827 PMC4739644

[7] Kulkarni AD , Jamieson DJ , Jones HW , et al. Fertility treatments and multiple births in the United States. N Engl J Med. 2013;369(23):2218‐2225. 24304051 10.1056/NEJMoa1301467

[8] Schieve LA , Meikle SF , Ferre C , Peterson HB , Jeng G , Wilcox LS . Low and very low birth weight in infants conceived with use of assisted reproductive technology. N Engl J Med. 2002;346(10):731‐737. 11882728 10.1056/NEJMoa010806

[9] Kissin DM , Kulkarni AD , Mneimneh A , et al. Embryo transfer practices and multiple births resulting from assisted reproductive technology: an opportunity for prevention. Fertil Steril. 2015;103(4):954‐961. 25637480 10.1016/j.fertnstert.2014.12.127 PMC4607049

[10] Vitthala S , Gelbaya TA , Brison DR , Fitzgerald CT , Nardo LG . The risk of monozygotic twins after assisted reproductive technology: a systematic review and meta‐analysis. Hum Reprod Update. 2009;15(1):45‐55. 18927071 10.1093/humupd/dmn045

[11] Knopman J , Krey LC , Lee J , Fino ME , Novetsky Akiva P , Noyes N . Monozygotic twinning: an eight‐year experience at a large IVF center. Fertil Steril. 2010;94(2):502‐510. 19409556 10.1016/j.fertnstert.2009.03.064

[12] Papanikolaou EG , Fatemi H , Venetis C , et al. Monozygotic twinning is not increased after single blastocyst transfer compared with single cleavage‐stage embryo transfer. Fertil Steril. 2010;93(2):592‐597. 19243755 10.1016/j.fertnstert.2008.12.088

[13] Sharara FI , Abdo G . Incidence of monozygotic twins in blastocyst and cleavage stage assisted reproductive technology cycles. Fertil Steril. 2010;93(2):642‐645. 19217097 10.1016/j.fertnstert.2008.12.130

[14] Kawachiya S , Bodri D , Shimada N , Kato K , Takehara Y , Kato O . Blastocyst culture is associated with an elevated incidence of monozygotic twinning after single embryo transfer. Fertil Steril. 2011;95(6):2140‐2142. 21215395 10.1016/j.fertnstert.2010.12.018

[15] Chang HJ , Lee JR , Jee BC , Suh CS , Kim SH . Impact of blastocyst transfer on offspring sex ratio and the monozygotic twinning rate: a systematic review and meta‐analysis. Fertil Steril. 2009;91(6):2381‐2390. 18718582 10.1016/j.fertnstert.2008.03.066

[16] Skiadas CC , Missmer SA , Benson CB , Gee RE , Racowsky C . Risk factors associated with pregnancies containing a monochorionic pair following assisted reproductive technologies. Hum Reprod. 2008;23(6):1366‐1371. 18378561 10.1093/humrep/den045

[17] Miura K , Niikawa N . Do monochorionic dizygotic twins increase after pregnancy by assisted reproductive technology[quest]. J Hum Genet. 2004;50(1):920‐925. 10.1007/s10038-004-0216-6 15599781

[18] Shalev SA , Shalev E , Pras E , et al. Evidence for blood chimerism in dizygotic spontaneous twin pregnancy discordant for Down syndrome. Prenat Diagn. 2006;26(9):782‐784. 16927328 10.1002/pd.1503

[19] Williams CA , Wallace MR , Drury KC , et al. Blood lymphocyte chimerism associated with IVF and monochorionic dizygous twinning: Case report. Hum Reprod. 2004;19(12):2816‐2821. 15375077 10.1093/humrep/deh533

[20] Herranz G . The timing of monozygotic twinning: a criticism of the common model. Zygote. 2015;23(01):27‐40. 23735171 10.1017/S0967199413000257

[21] McNamara HC , Kane SC , Craig JM , Short RV , Umstad MP . A review of the mechanisms and evidence for typical and atypical twinning. Am J Obstet Gynecol. 2016;214(2):172‐191. 26548710 10.1016/j.ajog.2015.10.930

[22] Gabbett MT , Laporte J , Sekar R , et al. Molecular support for heterogonesis resulting in sesquizygotic twinning. N Engl J Med. 2019;380(9):842‐849. 30811910 10.1056/NEJMoa1701313

[23] Daskalakis GJ , Nicolaides KH . Monozygotic twins discordant for body stalk anomaly. Ultrasound Obstet Gynecol. 2002;20(1):79‐81. 12100424 10.1046/j.1469-0705.2002.00631.x

[24] Chu S , Mao Q , Shapiro S , De Paepe ME . Placental endoglin levels in diamniotic‐monochorionic twin gestations: correlation with clinical and placental characteristics. Placenta. 2013;34(3):261‐268. 23306069 10.1016/j.placenta.2012.12.012

[25] Carlson BM . Human Embryology and Developmental Biology, 5th edn Philadelphia, PA: Elsevier/Saunders; 2014.

[26] Machin GA . Conjoined twins: implications for blastogenesis. Birth Defects Orig Artic Ser. 1993;29(1):141‐179. 8280870

[27] Kaufman MH . The embryology of conjoined twins. Childs Nerv Syst. 2004;20(8):508‐525. 15278382 10.1007/s00381-004-0985-4

[28] Barker DJ , Winter PD , Osmond C , Margetts B , Simmonds SJ . Weight in infancy and death from ischaemic heart disease. Lancet. 1989;2(8663):577‐580. 2570282 10.1016/s0140-6736(89)90710-1

[29] Ollikainen M , Smith KR , Joo EJ‐H , et al. DNA methylation analysis of multiple tissues from newborn twins reveals both genetic and intrauterine components to variation in the human neonatal epigenome. Hum Mol Genet. 2010;19(21):4176‐4188. 20699328 10.1093/hmg/ddq336

[30] Loke YJ , Galati JC , Morley R , et al. Association of maternal and nutrient supply line factors with DNA methylation at the imprinted IGF2/H19 locus in multiple tissues of newborn twins. Epigenetics. 2013;8(10):1069‐1079. 23917818 10.4161/epi.25908 PMC3891688

[31] Czyz W , Morahan JM , Ebers GC , Ramagopalan SV . Genetic, environmental and stochastic factors in monozygotic twin discordance with a focus on epigenetic differences. BMC Med. 2012;10(1):920‐12. 10.1186/1741-7015-10-93 PMC3566971 22898292

[32] Castillo‐Fernandez JE , Spector TD , Bell JT . Epigenetics of discordant monozygotic twins: implications for disease. Genome Med. 2014;6(7):60. 25484923 10.1186/s13073-014-0060-z PMC4254430

[33] Oates NA , van Vliet J , Duffy DL , et al. Increased DNA methylation at the AXIN1 gene in a monozygotic twin from a pair discordant for a caudal duplication anomaly. Am J Hum Genet. 2006;79(1):155‐162. 16773576 10.1086/505031 PMC1474116

[34] van Dongen J , Ehli E , Slieker R , et al. Epigenetic variation in monozygotic twins: a genome‐wide analysis of DNA methylation in buccal cells. Genes (Basel). 2014;5(2):347‐365. 24802513 10.3390/genes5020347 PMC4094937

[35] Mastroeni D , McKee A , Grover A , Rogers J , Coleman PD . Epigenetic differences in cortical neurons from a pair of monozygotic twins discordant for Alzheimer's disease. PLoS ONE. 2009;4(8):e6617. 19672297 10.1371/journal.pone.0006617 PMC2719870

[36] Clayton‐Smith J , Read AP , Donnai D . Monozygotic twinning and Wiedemann‐Beckwith syndrome. Am J Med Genet. 1992;42(4):633‐637. 1609846 10.1002/ajmg.1320420440

[37] Burn J , Povey S , Boyd Y , et al. Duchenne muscular dystrophy in one of monozygotic twin girls. J Med Genet. 1986;23(6):494‐500. 2879922 10.1136/jmg.23.6.494 PMC1049829

[38] Chutkow JG , Hyser CL , Edwards JA , Heffner Jr RR , Czyrny JJ . Monozygotic female twin carriers discordant for the clinical manifestations of Duchenne muscular dystrophy. Neurology. 1987;37(7):1147‐1151. 2885783 10.1212/wnl.37.7.1147

[39] Richards CS , Watkins SC , Hoffman EP , et al. Skewed X inactivation in a female MZ twin results in Duchenne muscular dystrophy. Am J Hum Genet. 1990;46(4):672‐681. 2180286 PMC1683658

[40] Tiberio G . MZ female twins discordant for X‐linked diseases: a review. Acta Genet Med Gemellol (Roma). 1994;43(3–4):207‐214. 8588495 10.1017/s0001566000001963

[41] Zeng SM , Yankowitz J , Murray JC . Conjoined twins in a monozygotic triplet pregnancy: prenatal diagnosis and X‐inactivation. Teratology. 2002;66(6):278‐281. 12486760 10.1002/tera.10091

[42] Vadlamudi L , Dibbens LM , Lawrence KM , et al. Timing of de novo mutagenesis — a twin study of sodium‐channel mutations. N Engl J Med. 2010;363(14):1335‐1340. 20879882 10.1056/NEJMoa0910752

[43] Yadav SK , Kumari A , Javed S , Ali S . DYZ1 arrays show sequence variation between the monozygotic males. BMC Genet. 2014;15(1):920‐12. 10.1186/1471-2156-15-19 PMC3925983 24495361

[44] Li R , Montpetit A , Rousseau M , et al. Somatic point mutations occurring early in development: a monozygotic twin study. J Med Genet. 2014;51(1):28‐34. 24123875 10.1136/jmedgenet-2013-101712

[45] Haque FN , Gottesman II , Wong AH . Not really identical: epigenetic differences in monozygotic twins and implications for twin studies in psychiatry. Am J Med Genet C Semin Med Genet. 2009;151C(2):136‐141. 19378334 10.1002/ajmg.c.30206

[46] Abdellaoui A , Ehli EA , Hottenga J‐J , et al. CNV concordance in 1,097 MZ twin pairs. Twin Res Hum Genet. 2015;18(01):920‐12. 10.1017/thg.2014.86 25578775

[47] Bourthoumieu S , Yardin C , Terro F , et al. Monozygotic twins concordant for blood karyotype, but phenotypically discordant: a case of "mosaic chimerism". Am J Med Genet A. 2005;135(2):190‐194. 15832362 10.1002/ajmg.a.30674

[48] Chaiyasap P , Kulawonganunchai S , Srichomthong C , Tongsima S , Suphapeetiporn K , Shotelersuk V . Whole genome and exome sequencing of monozygotic twins with trisomy 21, discordant for a congenital heart defect and epilepsy. PLoS ONE. 2014;9(6):e100191. 24950249 10.1371/journal.pone.0100191 PMC4064986

[49] Petersen B‐S , Spehlmann ME , Raedler A , et al. Whole genome and exome sequencing of monozygotic twins discordant for Crohn’s disease. BMC Genomics. 2014;15(1):920‐11. 24996980 10.1186/1471-2164-15-564 PMC4102722

[50] Morin L , Lim K ; Diagnostic Imaging Committee ; Special Contributor ; Genetics Committee ; Maternal Fetal Medicine Committee . Ultrasound in twin pregnancies. J Obstet Gynaecol Can. 2011;33(6):643‐656. 21846456 10.1016/S1701-2163(16)34916-7

[51] Winsor EJ , Akoury H , Chitayat D , Steele L , Stockley TL . The role of molecular microsatellite identity testing to detect sampling errors in prenatal diagnosis. Prenat Diagn. 2010;30(8):746‐752. 20661888 10.1002/pd.2530

[52] Blickstein I , Keith LG . Multiple Pregnancy: Epidemiology, Gestation, and Perinatal Outcome. Boca Raton, FL: CRC Press; 2005.

[53] Gil MM , Quezada MS , Bregant B , Syngelaki A , Nicolaides KH . Cell‐free DNA analysis for trisomy risk assessment in first‐trimester twin pregnancies *.* Fetal Diagn Ther. 2013;35(3):204‐211. 24247435 10.1159/000356495

[54] van Jaarsveld C , Llewellyn CH , Fildes A , Fisher A , Wardle J . Are my twins identical: parents may be misinformed by prenatal scan observations. BJOG. 2012;119(5):517‐518. 22372493 10.1111/j.1471-0528.2012.03281.x

